# Requirements and special considerations for drug trials with children across six jurisdictions: 1. Clinical trial application review in the regulatory approval process

**DOI:** 10.3389/fmed.2025.1542408

**Published:** 2025-04-15

**Authors:** Breanne Stewart, Pirkko Lepola, Gunter F. Egger, Fahimeda Ali, Albert J. Allen, Alysha K. Croker, Andrew J. Davidson, Pamela Dicks, Saul N. Faust, Dionna Green, Collin Hovinga, Agnes V. Klein, Robyn Langham, Hidefumi Nakamura, Laura Pioppo, Shiva Ramroop, Michiyo Sakiyama, Isabel Sanchez Vigil de la Villa, Junko Sato, Donna L. Snyder, Mark A. Turner, Sarah Zaidi, Kanecia Zimmerman, Thierry Lacaze-Masmonteil

**Affiliations:** ^1^Maternal Infant Child and Youth Research Network (MICYRN), Vancouver, BC, Canada; ^2^Department of Children and Adolescents, University of Helsinki and Helsinki University Hospital, Helsinki, Finland; ^3^Pediatric Medicine Office and Data Analytics and Methods Task Force, European Medicines Agency (EMA), Amsterdam, Netherlands; ^4^Paediatrics—Innovative Medicines, Healthcare Quality & Access, MHRA, London, United Kingdom; ^5^Institute for Advances Clinical Trials for Children, Rockville, MD, United States; ^6^Critical Path Institute, Tucson, AZ, United States; ^7^Biologic and Radiopharmaceutical Drugs Directorate, Health Products and Food Branch, Health Canada, Ottawa, ON, Canada; ^8^Melbourne Children's Trials Centre, Murdoch Children's Research Institute, Melbourne, VIC, Australia; ^9^NHS Scottish Children's Research Network, Royal Aberdeen Children's Hospital, Aberdeen, United Kingdom; ^10^NIHR Southampton Clinical Research Facility and Biomedical Research Centre, University Hospital Southampton NHS Foundation Trust, Southampton, United Kingdom; ^11^Faculty of Medicine and Institute for Life Sciences, University of Southampton, Southampton, United Kingdom; ^12^Office of Pediatric Therapeutics, Office of the Commissioner, United States Food and Drug Administration, Silver Spring, MD, United States; ^13^Therapeutic Goods Administration, Australian Government Department of Health and Aged Care, Canberra, ACT, Australia; ^14^Department of Research and Development Supervision, National Centre for Child Health and Development, Tokyo, Japan; ^15^Pediatric Drug Working Group, Pharmaceuticals and Medical Devices Agency, Tokyo, Japan; ^16^WCG Clinical, Princeton, NJ, United States; ^17^Department of Women's and Children's Health, University of Liverpool, Liverpool, United Kingdom; ^18^Conect4children Stichting, Utrecht, Netherlands; ^19^Duke Clinical Research Institute, Duke University School of Medicine, Durham, NC, United States; ^20^Department of Pediatrics, Duke University School of Medicine, Durham, NC, United States

**Keywords:** pediatrics, clinical trials, regulatory science, clinical trial application, clinical trial authorization, risk-based approach

## Abstract

**Background:**

Conducting clinical trials (CTs) with children presents several challenges. A major challenge is the need to enrol participants at multiple sites across different jurisdictions. Regardless of whether the trials involve children, adults, or both, CTs need to meet separate Competent Authority (CA) requirements to proceed in each participating country. This work, undertaken by the Working Group (WG) on International Collaborations at the European Network of Pediatric Research at the European Medicines Agency (Enpr-EMA) aims to describe the regulatory requirements including any specific to pediatrics, as well as current or upcoming changes across six jurisdictions—the European Union (EU), United Kingdom (UK), United States of America (USA), Canada, Japan, and Australia.

**Methods:**

An open questionnaire developed by the WG and directed at both the CA and the national pediatric clinical trial networks arranged by jurisdictions.

**Results:**

A synopsis of the current legislative and regulatory requirements for CTs applications, application submission processes and application requirements is presented for each of the six jurisdictions. Requirements were found to be mostly consistent across jurisdictions. No difference was found in processes for CTs submission, review, and authorization for pediatric CTs vs. CTs in adults. However, there are additional Ethics Committee/Institutional Review Board requirements for clinical trials including children. Some jurisdictions are considering adopting a risk-based approach, inspired by the Organization of Economic Co-operation and Development (OECD) recommendations on Governance. Changes currently or soon to be implemented in some jurisdictions are also described.

**Conclusions:**

Regulators from the jurisdictions represented in this WG are collaborating to facilitate regulatory harmonization and foster international alignment of pediatric CTs. By interacting with their respective regulatory bodies and developing expertise in their jurisdiction's regulatory requirements, national pediatric networks can support both academic and industry sponsors in navigating the regulatory process for CTs.

## 1 Introduction

Economic, ethical, regulatory and infrastructure considerations related to child health research have limited the number of drug clinical trials (CTs) conducted with children (1–3). There are several challenges, more frequently encountered when conducting CTs with a pediatric population, that have contributed to current gaps in the evidence base on medicines for children. Many diseases occur less frequently in children and neonates, compared to adults. Conversely, some diseases only occur in children or have a different etiology and/or pathophysiology from that of the adult population. Other challenges, both with neonatal and pediatric CTs include the frequent lack of age-appropriate and validated clinical endpoints and pharmacokinetic data, the paucity of data on reference laboratory values, and the need for sensitive assays to minimize blood sampling. While children cannot legally provide consent to participate in CTs, they should both be informed with age-appropriate information and have a voice regarding their participation (assent) when they are capable of doing so (4–6). Therefore, in order to engage a sufficient number of participants in a timely fashion and to execute well and appropriately powered studies, pediatric CTs often require recruitment and enrollment in multiple countries as well as across multiple sites and jurisdictions. Expanding studies across multiple jurisdictions requires considerable effort and may necessitate specific procedures. There is also a complexity due to the need to obtain clearance from the Competent Authority (CA), negotiate budgets, execute contracts and data sharing agreements, and undergo review by institutional ethics committees (EC) or Institutional Review Board (IRB).

CTs need to meet separate CA and at least one (and often multiple) EC/IRB requirements in each participating jurisdiction, regardless of the population investigated (children, adults, or combination of both). Whether the CT is industry-sponsored or investigator-initiated, time and resource investments must be spent in developing documentation and correspondence for regulatory submissions to authorize CTs (7). To streamline this process, several jurisdictions have recently modernized or are in the process of modernizing their legislation and/or regulations for clinical trial application (CTA) submission and review. This work aims to describe the regulatory and ethics requirements around CTs, including any pediatric specific requirements, as well as current or upcoming changes across six jurisdictions: the European Union (EU), United Kingdom (UK), United States of America (USA), Canada, Japan, and Australia. The article has three main objectives: to assist investigators and industry sponsors in conducting multi-jurisdictional CTs in children, to identify regulatory and ethical challenges in conducting these trials on an international scale and to foster and enhance international collaboration.

This first article focuses on the Clinical Trial Application (CTA), known as the Investigational New Drug Application (IND) in the USA, and the respective jurisdictional requirements from a child health perspective. An accompanying article in the same issue of this journal describes the similarities and differences as well as the jurisdiction-specific guidance for the research ethics review process.

## 2 Materials and methods

In 2007, the Pediatric Cluster was established by the European Medicines Agency (EMA) in the EU and the US Food and Drug Administration (FDA), as a forum to discuss the approach to pediatric development, pertaining to specific products, product classes or therapeutic areas to enhance the science of pediatric CTs and inform the pediatric development plans submitted to both agencies. Over the following years, the Pharmaceuticals and Medical Devices Agency (PMDA), Japan, Health Canada (HC), and the Therapeutic Goods Administration (TGA), Australia, joined the Pediatric Cluster. The five CAs meet on at least a monthly basis to discuss all aspects of pediatric development, including trial design, ethics, safety, and pediatric study feasibility. In 2018, building on this long standing international collaborative exchange, the European Network of Paediatric Research at the European Medicines Agency (Enpr-EMA) established a Working Group (WG) on international collaborations made of the same five CAs and the national pediatrics clinical trials networks of the corresponding jurisdictions with the specific aim to facilitate the unique needs of pediatric studies in a multi-country, multisite and multijurisdictional setting. In addition, after the United Kingdom (UK) left the EU on the 31st of January 2020, the UK regulator (Medicines and Healthcare Products Regulatory Agency, MHRA) and two UK pediatric research national networks joined the working group.

The following networks participated in the survey:

- EU: Enpr-EMA and Conect4Children (one of the Enpr-EMA member networks).
- USA: Pediatric Trials Network at Duke University and I-ACT (Institute for Advanced Clinical Trials in Children).
- Australia: Australian Network of Paediatric Trial Centres (ANPTC).
- Japan: Japanese Pediatric Society Drug Development Network (JPedNet).
- Canada: Maternal Infant Child and Youth Research Network (MICYRN).
- UK: (National Institute for Health Research Clinical Research Network-Children, (NIHR CRN-Children), and the National Health Services Scottish Children's Research Network, (NHS Sco CRN).

The WG conducted an environmental scan across jurisdictions using an open questionnaire directed at both the CA and the networks organized by jurisdictions under the four following headings:

- Clinical trial regulatory requirements.
- Clinical trial application submission process.
- Clinical trial application requirements: Organization for Economic Co-operation and Development (OECD) Recommendation Status (i.e., use of different requirements based on the actual risk associated with the CT, in order to both facilitate International CT and streamline procedures for low/intermediate risk CT) (8).
- Clinical trial site: Ethics requirements (results presented in accompanying article).

The topics surveyed—regulatory requirements, application submission process, OECD risk-based classification, and ethics requirements—were chosen to address the core elements of clinical trial authorization processes across different jurisdictions. Including these areas enabled the working group to analyze critical regulatory and procedural similarities and differences that could impact the implementation and harmonization of pediatric clinical trials internationally. Insights gained from the survey responses could help inform strategies for streamlining cross-border approvals, and assess the feasibility of adopting risk-based approaches to regulation. It took ~2 years to all CAs and Networks to complete and obtain the survey (2020/21). The delay in survey completion was the result of the high-level clearance that was required from the CAs for some of the jurisdictions, compounded by the fact that the survey was being completed in the initial parts of the COVID-19 pandemic. After the UK left the EU, the same questionnaire was completed by the UK regulator (MHRA) and the two UK Pediatric research national networks. All responses were reviewed together by the respective jurisdiction's CA and pediatric trial network(s) to ensure agreement in responses. In drafting this manuscript, participants were repolled (2024) to confirm any new updates or changes in status of planned updates to their jurisdiction's practices.

For each of the categories listed above, a summary of the status is provided, as well as changes currently being implemented or to be implemented in the near future.

## 3 Results

### 3.1 Clinical trial regulatory requirements

Clinical trials with children must meet rigorous regulatory standards, often similar across jurisdictions. Although countries may have unique legislative frameworks, most have protocols that define investigational product status, govern the authority responsible for oversight, and require ethics review. International regulatory alignment, especially among major jurisdictions, is facilitated by the Pediatric Cluster and efforts to standardize pediatric clinical trial requirements. By understanding these regulatory frameworks, sponsors and researchers can streamline trial approval processes across borders.

A synopsis of the current legislative/regulatory requirements for CTAs, investigational status determination, and body(ies) responsible for determining investigational status is provided in Table 1.

**Table 1:** Key clinical trial regulatory requirement.

	**Legislation/regulation: clinical trials**	**Proposed future change to legislation/regulation**	**Investigational status determination**	**Body(ies) responsible for determining if a CTA/IND is needed**	**Circumstances where CTA/IND is not required**
**Australia**	Therapeutic Goods Act and Regulations GCP Guidelines; National Statement on Ethical Conduct of Clinical Trials; HREC approved protocol and amendments	No	Any therapeutic good (medicine, device, biological) not entered into the Australian Register of Therapeutic Goods (ARTG); Any therapeutic good listed in ARTG used beyond the conditions of its marketing	The choice of which scheme to use (Clinical Trial Notification (CTN) or CTA) lies firstly with the trial sponsor and then with the HREC that approves the protocol. The determining factor for a HREC is whether the committee has access to appropriate scientific and technical expertise in order to assess the safety of the product and if the product falls under the mandatory requirements of the CTA	The CTA scheme is mandatory for class 4 biologicals unless an earlier clinical trial has checked the safety of the unapproved therapeutic good, or a national regulatory body with comparable regulatory requirements (for example, the FDA) has approved a clinical trial for an equivalent indication
**Canada**	The food and drug regulations: part C, division 5 (drugs and biologics)	Health Canada is currently reviewing its regulations for clinical trials with drugs, devices, and natural health products to adopt a more risk-based approach	Drugs that have not been authorized for sale in Canada; Marketed drugs where the proposed trial is outside of the parameters of the drug's marketed status in Canada: indication and clinical use, target patient population, route of administration, or dosage regimen	Health Canada	Observational studies; A drug that has received a notice of compliance (NOC, “marketed drug”) and is being used within the parameters of its authorized use
**EU**	Regulation (EU) No 536/2014 of the European Parliament and of the Council, implemented in the EU member states on 31 January 2022		Investigational medicinal product means a medicinal product which is being tested or used as a reference, including as a placebo, in a clinical trial.	Each Member State determines the appropriate bodies to be involved.	No
**UK**	The Medicines for Human Use (Clinical Trials) Regulations 2004	The UK is currently working to develop proposals to improve the regulatory framework for clinical trials	“Investigational medicinal product” means a pharmaceutical form of an active substance or placebo being tested, or to be tested, or used, or to be used, as a reference in a clinical trial, and includes a medicinal product which has a marketing authorization but is, for the purposes of the trial—(a)used or assembled (formulated or packaged) in a way different from the form of the product authorized under the authorization, (b)used for an indication not included in the summary of product characteristics under the authorization for that product, or (c)used to gain further information about the form of that product as authorized under the authorization	Medicines and Healthcare products Regulatory Agency (MHRA)	Legislation only applies to interventional clinical trials. A definition of a non-interventional trial is included in UK legislation
**Japan**	Pharmaceutical and Medical Device Act (PMDA); All ICH GLs such as E11, E6 are implemented as regulations.	Five years have passed since the implementation of the Clinical Trials Act (Act No. 16 of April 14, 2017). A revision is planned.	Non-authorized medications; Marketed drugs where the proposed trial is outside of the parameters of the marketed status: New ingredient, new route of administration, or new combination	Pharmaceuticals and Medical Device Agency (PMDA)	Clinical trials not for regulatory approval
**USA**	Best Pharmaceuticals for Children Act (BPCA) and the Pediatric Research Equity Act (PREA)	No	For a drug, this determination is based on part 312 of Title 21 of the Code of Federal Regulations (CFR) for Food and Drugs. These regulations define an investigational drug, in relevant part, as “a new drug or biological drug that is used in a clinical investigation”	The potential sponsor of a planned clinical investigation using a marketed drug is responsible for determining whether the investigation meets the criteria for an exemption and can seek advice from the Food and Drug Administration (FDA) if there is uncertainty about whether IND exemption criteria are met	Criteria that must be met to be exempt from IND requirements are specified in 21 CFR 312.2(b), 21 CFR 320.31, and 21 CFR 361.1. These criteria are discussed in FDA's “Guidance for Clinical Investigations, Sponsors and IRBs Investigational New Drug Applications- Determining Whether Human Research Studies Can Be Conducted Without An IND”

### 3.2 Clinical trial application submission review process

The clinical trial application (CTA) review process varies slightly by jurisdiction but generally involves timelines for initial review, amendment approvals, and specific responses from regulatory authorities. Understanding these submission processes, is essential for efficient trial setup.

Table 2 provides a summary of the current review process in each jurisdiction including body(ies) responsible for review, timelines, responses issued by the CA regarding the review, and post authorization obligations.

**Table 2:** Submission process for clinical trial application.

**Country**	**Body responsible for reviewing pediatric CTA/IND**	**Regulatory review timeline for CTA/IND (Number of Days)**	**Regulatory review timeline for CTA/IND Amendment (Number of Days)**	**Responses issued by regulatory authority for study**	**Obligations mandated by regulatory body(ies) during conduct of CT**
**Australia**	HREC	CTA—target timeframe is 50 days; CTN—does not apply. Notifications processed in5 days	CTA—50 days; CTN—processed in 5days	CTA—approval or rejection letter; CTN—trial notified and paid, sponsor can start the trial	As required by ICH-GCP and protocol
**Canada**	Health Canada; Pharmaceutical Drugs Directorate (PDD) for pharmaceutical drugs; Biologic and Radiopharmaceutical Drugs Directorate (BRDD) for radiopharmaceuticals, biologics, cell, and gene therapies	30 days commenced on the date of receipt of a completed application	30 days	No Objection Letter (NOL) - Sponsor may proceed with the proposed trial; Not Satisfactory Notice (NSN)—Sponsor may not proceed with the proposed trial as regulatory requirements are not met	Notification to Health Canada of a discontinuation of a trial, resumption of a trial, completion of a trial and closure of sites. Any rejection/negative decision of protocol by Research Ethics Boards; Safety reporting of serious, unexpected adverse drug reactions (ADR) that occurred both inside and outside of Canada.
**EU**	EU Member States will determine appropriate bodies to be involved in the revision, organizing also Ethics Committees participation. The CTA dossier will be made of Part I and Part II data and documents. Evaluation of Part I of the dossier comprises a coordinated assessment between Member States Concerned (MSC) and assessment of Part II involves individual evaluation by each MSC. After assessment of Part I and Part II, a single decision is issued by each MSC	45 assessment days commenced on the day of receipt of valid application (extended maximum timeframes possible as per legislation for Advanced Therapy Medicinal Products (ATMPs) or if communication with sponsor is needed to resolve issues); Assessment is followed by maximum 5-day decision phase	38 assessment days commenced on the day of receipt of valid application (extended maximum timeframes possible as per legislation for Advanced Therapy Medicinal Products (ATMPs) or if communication with sponsor is needed to resolve issues); Assessment is followed by maximum 5-day decision phase	The CTA is authorized or authorized subject to compliance with specific conditions or not authorized	All information generated during and after the lifecycle of the trial, including for example start of trial, start of recruitment, end of trial, summary of results must be submitted to the EU portal database
**UK**	Medicines and Healthcare products Regulatory Agency (MHRA)	Maximum 30 days commenced on the day of receipt for initial review (maximum 60 days if communication with sponsor needed to resolve issues) Certain products (e.g., advanced therapy) may have extended maximum timeframes as per legislation	35 days maximum	Authorized; Authorized subject to specific conditions; Not authorized	Sponsors are required to submit annual safety reports, suspected unexpected serious adverse reactions (SUSARs) and urgent safety measures taken; Notification required when the trial has ended
**Japan**	Pharmaceutical and Medical Device Act (PMDA)	30 days commenced on the day of receipt for initial CT notification	N/A	If no response is received within 30 days for first clinical trial notification of the development and 14 days for others trials. When submission is not acceptable, NCA require the sponsor to revise the protocol	Sponsor is required to report the followings to PMDA: discontinuation of a trial, resumption of a trial or completion/closure of sites. Sponsors are required to submit annual safety reports, suspected unexpected serious adverse reactions and urgent safety measures taken. Safety reporting of adverse drug reactions (ADR). Updating the investigator brochure
**United States**	FDA	Up to 30 days for the initial IND 120 days for a PPSR submitted under BPCA and 210 days for an initial PSP (iPSP)	No waiting period for IND amendments although new protocols and protocol changes to ongoing trials require prior approval by an IRB unless the change to the protocol is necessary to eliminate apparent immediate hazards to human subjects Review of a revised PPSR is 120 days; Review of an amended iPSP is 210 days	INDs: An IND goes into effect 30 days after FDA receives the IND (21 CFR 312.40), unless FDA notifies the sponsor that the investigations described in the IND are subject to a clinical hold under § 312.42; or on earlier notification by FDA that the clinical investigations in the IND may begin. Clinical hold: Applicable to an initial IND submission, an ongoing clinical investigation, or resumption of clinical investigations that have been placed on clinical hold (i.e., the clinical hold has been lifted), inactive status or terminated. PPSRs submitted under BPCA: (1) Issue a Written Request (WR); (2) Issue an inadequate letter For iPSPs submitted under PREA: (1) Agreed (2) Non-agreed (3) Materially incomplete	IND safety reporting is outlined in 21CFR 312.32 including definitions, review of safety information, IND safety reports and follow up. Annual reporting criteria are outlined in 21 CFR 312.33. 21 CFR 314.80 outlines post-marketing reporting of adverse drug experiences, including definitions, review of adverse drug experiences, reporting requirements, scientific literature, post-marketing studies, information reported on individual case safety reports, electronic format for submissions, multiple reports, patient privacy, recordkeeping, and withdrawal of approval

### 3.3 Trial application requirement according to OECD risk-based classification

Jurisdictions increasingly adopt risk-based classifications, as recommended by the OECD, to simplify regulatory requirements for low-risk trials. This approach can reduce burdens on sponsors by scaling requirements based on the investigational product's risk level and marketing status. For instance, low-risk trials of marketed drugs might benefit from relaxed labeling and monitoring rules. This classification facilitates broader access to safe and effective pediatric treatments while maintaining necessary safeguards.

Table 3 summarizes the current status for each jurisdiction in regard to the OECD risk-based classification. As described below, some jurisdictions are considering adopting a risk-based approach inspired by the OECD recommendation on the Governance of CTs (8).

**Table 3:** Clinical trial application requirements based on OECD recommendation status.

**Country**	**Non-authorized medications**	**Authorized medications used outside marketing authorization parameters-not supported by established medical practice**	**Authorized medications used outside marketing authorization parameters-supported by established medical practice**	**Authorized medications tested within marketing authorization**
**Australia**	Yes	Yes	Yes	No
**Canada**	Yes	Yes	Yes	No
**EU**	Yes	Yes	Yes- less stringent requirements for low-intervention trials as regards monitoring, requirements for the contents of the master file and traceability of investigational medicinal products.	No
**UK**	Yes	Yes	Yes—via a “notification scheme”. https://www.gov.uk/guidance/clinical-trials-for-medicines-apply-for-authorisation-in-the-uk#notification-scheme Risk adaption allowed	No (non-interventional). Yes, if interventional via a ‘notification scheme'. Risk adaption allowed
**Japan**	Yes	Yes	Yes	No
**United States**	Yes, if the research meets the definition of a drug and a clinical investigation as defined by FDA's regulations and the clinical investigation is not exempt from the IND requirements	Yes, unless the clinical investigation meets the criteria for an exemption from the IND requirements	Yes, unless the clinical investigation meets the criteria for an exemption from the IND requirements	No (provided the intent is not to support a significant change in the pediatric labeling of the drug)

### 3.4 Changes recently, currently, or soon to be implemented in some jurisdictions

Several jurisdictions are modernizing clinical trial regulations to improve efficiency and adaptability. For instance, the EU has implemented the Clinical Trials Information System (CTIS), and Health Canada is exploring a proportional risk-based framework. In the UK, combined review processes expedite ethics and regulatory approvals. These changes aim to support innovative trial designs, enable rapid responses to public health needs, and encourage global trial harmonization, ultimately fostering faster access to pediatric treatments.

#### 3.4.1 European Union

In line with the aim of harmonizing clinical trial regulations across jurisdictions and in an effort to facilitate the involvement of multiple regions and sites, the EU has recently reviewed the existing provisions governing clinical trials contained in Directive 2001/20/EC, and has adopted Clinical Trial Regulation (EU) No 536/2014 which promotes efficiency in terms of CTA revision timelines, a focus on patients and specificities of the different populations, transparency of data on clinical research, and mutual collaboration between all EU Member States (MSs) and European Economic Area (EEA) countries (6). The Regulation covers 30 EEA countries; the 27 EU MS together with the European Free Trade Association countries Iceland, Liechtenstein, and Norway (9). Each country has the responsibility to evaluate, authorize and oversee clinical trials. However, the Regulation harmonizes throughout these countries the processes for assessment and oversight of both commercial and non-commercial CTs, via the Clinical Trials Information System (CTIS), the single EU portal with a database (10).

To streamline and facilitate the flow of information between sponsors and EEA/EU MSs, the EU portal CTIS, has been established to be used as a single-entry point for the submission of CTA data and information, that will also be stored and publicly available in the database. The process for submitting, reviewing, and authorizing CTs has been simplified by the submission of one application dossier, including two parts (I and II) to all countries concerned through the CTIS obtaining a single decision. Each country determines according to their internal organization, the appropriate competent authority (CA), and ethics bodies (EC) to be involved in the assessment of the application in the CTIS. Moreover, assessment reports and final decisions are submitted to the sponsors through the CTIS.

The CTA dossier and the related assessment reports in the CTIS are divided into two parts. Part I comprises more general information on the trial, evaluating among other features the trial protocol, trial design, the benefit and risk balance, information on the Investigational Medicinal Products (IMP) being used in the trial, including Investigator Brochure and the IMP Dossier.

The assessment of Part I of the application is conducted in a coordinated manner between Member States Concerned (MSCs) and as a result one consolidated conclusion is issued by the MSC in the lead of Part I assessment, namely the Reporting Member State (RMS). Part II includes information considered to be specific for each MSC, mainly concerning the ethics review aspects related to the protection of subjects (11). Therefore, Part II of the application is assessed by each MSC for its own territory, resulting in an individual conclusion for each MSC. After completion of the assessment of Part I and Part II, every MSC notifies the sponsor on whether the clinical trial application is authorized fully, subject to conditions or, if authorization is refused, by way of issuing one single decision comprising the joint conclusion of Part I assessment report, along with the individual conclusions for Part II assessment reports. The EU CTIS has a key role providing the business tool to permit mutual interactions and communication throughout the evaluation of the CTA and during the entire clinical trial life cycle.

The EU Regulation was signed in 2014 and came into force in 2016, but it was applied fully on the 31st of January 2022, when the EU portal and the EU database (CTIS) had achieved full functionality and the systems met the structural specifications. A three-year transition period, which was designed to ensure a smooth repeal of the EU Clinical Trials Directive (EC) No. 2001/20/EC and any national legislation that had been put in place as part of the transposition of the Directive, ended on 31st of January 2025.

The results of pediatric clinical trials, whether positive or negative, must be submitted to the EMA through CTIS within 6 months of trial completion to ensure public access to pediatric research data. All clinical trials in EU/EEA previously submitted through the European Union Drug Regulating Authorities Clinical Trials Database (Eudra-CT: https://eudract.ema.europa.eu/) between 1 May 2004 until 30 January 2023 under the Directive are still visible in the EU Clinical Trials Register (https://www.clinicaltrialsregister.eu/) as the EU CTR continues to display information on EudraCT trials.

In the EU, the development of pediatric medicines is governed by the Pediatric Regulation (EC) No. 1901/2006, which came into effect in 2007 and requires that companies submit study results in children according to an agreed Pediatric Investigation Plan (PIP) for new medicines unless a waiver or deferral is granted (12). The same requirement also applies when a marketing-authorization holder wants to add a new indication, pharmaceutical form or route of administration for a medicine that is already authorized and covered by intellectual property rights. The PIP ensures that medicinal products are appropriately studied in children, with the aim of increasing the availability of pediatric-specific data and formulations. The PIP must be agreed upon with the EMA's Pediatric Committee (PDCO) before a company can submit a Marketing Authorization Application (MAA) in the EU, and it outlines the studies necessary to support the pediatric use of a medicine, ensuring that they are conducted at the appropriate stages of development. Deferrals can be granted when it is e.g., not feasible or safe to conduct pediatric studies at the same time as adult studies, allowing data to be collected later. Completion of the PIP in compliance with its agreed elements can result in rewards, including a 6-month extension of the Supplementary Protection Certificate (SPC), and a 2-year extension of market exclusivity for orphan medicines. However, it is important to know that clinical trials which include patients under 18 years of age can also be conducted if they are not part of an agreed PIP, provided they receive regulatory and ethics board approval.

To improve the flexibility of the PIP process and address the challenges of early-stage pediatric planning, the EMA introduced the stepwise PIP Pilot in 2022 (13). This pilot program allows sponsors in certain cases to submit an initial PIP even if not all elements are known yet, with the option to update and refine the plan in a stepwise manner as additional clinical and non-clinical data become available. The aim is to enable a more iterative and data-driven approach to pediatric drug development, aligning regulatory expectations with the evolving understanding of a medicine's safety and efficacy in children. The pilot enhances regulatory dialogue between sponsors and the PDCO and is also intended to promote greater alignment between global regulatory authorities, including the FDA, to support international collaboration in pediatric drug development.

In April 2023, the European Commission published a proposal for a new pharmaceutical legislation, which retains the obligations and rewards for pediatric study plans but introduces several changes, including integrating the stepwise PIP into the regulatory framework (14). The proposed revisions aim to increase regulatory flexibility, reduce delays in pediatric drug development, and better align incentives to encourage investment in pediatric research. If adopted, these changes would modernize the existing framework while continuing to prioritize the development of safe and effective medicines for children.

Building on the application of the Clinical Trial Regulation, in January 2022 the European Commission (EC), the Heads of Medicines Agencies (HMA) and the EMA launched an initiative which aims to further strengthen the European environment for clinical trials [Accelerating Clinical Trials in the EU (ACT EU)]. This initiative includes actions to further promote clinical research such as publishing guidance focused on complex, innovative and decentralized trials, establishing a multi-stakeholder platform that includes patients, and evaluating the implementation of the Regulation (15).

#### 3.4.2 Health Canada clinical trials modernization initiative

Recent evolutions in the clinical trial environment including, for example, new CT designs for diseases where trial enrollment is expected to be challenging (pediatric diseases, rare diseases), risk management options, greater complexity of CT designs, global alignment and new uses of both data and technology have necessitated the modernization of Canada's current clinical trial regulations to ensure people in Canada have timely access to safe and efficacious medicines. The following key changes have been proposed as part of Health Canada's Clinical Trial Modernization Initiative: (a) proportional risk-based approach to regulation; (b) single authorization of a trial involving multiple arms, with the ability to suspend or cancel an authorization in part or in whole; (c) agile lifecycle across the conduct of the trial, with the ability to add terms and conditions when appropriate; (d) enabling decentralized trials; and (e) new policy for registration and summary results reporting, along with a new search portal to facilitate public access to Canadian clinical trials. Flexible and risk-based regulations, as proposed by the OECD international framework, are expected to allow for more trials and greater representation of children in national and global trials (8). Additionally, by aligning with international risk-based frameworks and enabling novel trial design, the availability and accessibility of new drugs for people in Canada could increase (16).

While Health Canada requires drugs to be authorized in order to be marketed for specific uses in Canada, clinical trials may involve the off-label use of a marketed drug (i.e., outside the authorized purpose or condition of use). Additionally, in some cases, the off-label uses of an authorized drug in a trial may be consistent with the standard of medical practice overseen by provincial and territorial bodies that govern medical practice. Health Canada is proposing to introduce some flexibilities for such drugs used in the CT which would exempt sponsors from certain requirements, such as CT-specific labeling or some record keeping requirements (17).

#### 3.4.3 United Kingdom

For many years, the UK has had a single regulatory application process for regulations, Medicines and Healthcare Products Regulatory Agency (MHRA) and ethics (Health Research Authority National Ethics Service) multicenter CTAs, although each agency acted independently in their review. Having left the EU, the UK updated processes and designed a national regulatory environment for CTs to support the development of innovative medicines for the benefit of patients and public health. The MHRA worked with the Health Research Authority (HRA) and stakeholder partners including patients, academics, charities, pharma and contract research organizations, to review the existing UK legislation and explore how it might be best updated to support patients and the UK clinical research sector. Consideration was given on ways to streamline CTA approvals, enable innovation, enhance CT transparency, better define risk proportionality, and promote patient and public involvement in CTs. An additional aim of the proposals was to ensure the legislation builds international interoperability thereby supporting the UK's role as a key hub to conduct multi-national trials. The output of this exercise informed legislative proposals that were published in an 8-week public consultation on 17 January 2022 (18). The consultation closed on 14 March 2022 and at time of writing the UK government response to feedback was under preparation.

This opportunity forms part of a coordinated and coherent program of work that was developed in 2022 to ensure the Recovery, Resilience and Growth (RRG) of UK clinical research delivery set out in the vision for the future of clinical research delivery (19).

A key objective of the RRG program was even more streamlined, efficient and innovative clinical research. As part of this effort a revised “combined review” service was established by the MHRA and the HRA and has involved collaborative working with the National Institute for Health and Care Research (NIHR) and the devolved administrations. The refreshed combined review service offers a single application submission route and a coordinated review for CTs of investigational medicinal products, resulting in a combined regulatory and ethics decision on a clinical trial. From January 2022, this is the route all new UK clinical trials are submitted and reviewed. It is anticipated this will facilitate faster approval times than via the previous separate systems and speedier set up (20). The MHRA will continue to make enhancements with new legislative measures to make it easier and faster for applicants to gain approvals and to ensure the UK remains a prime destination for clinical trials (21). More detailed information on the planned overhaul of UK clinical trials regulations were recently (December 2024) made available (22).

#### 3.4.4 United States

A brief recap of the IND process and some recent FDA initiatives that impact pediatric CTA submissions are described here.

In the USA, an IND application is required if an investigator or sponsor plans to administer to children in a CT a drug that is not approved for use in children unless the drug/biologic meets criteria for an IND exemption (23). Exemptions from IND requirements are outlined in 21 CFR 312.2, 21 CFR 320.31, and 21 CFR 361.1 (24). The three most commonly occurring scenarios when clinical investigations may be exempted from the IND application requirements are certain clinical investigations of lawfully marketed drugs, certain bioavailability or bioequivalence studies, or clinical investigations involving certain uses of radioactive drugs generally considered as safe and effective for those uses (24). For each of these and few other scenarios, the specific criteria for exemption described in the regulations must be met.

The Pediatric Research Equity Act (PREA) and Best Pharmaceuticals for Children Act (BPCA) are important drivers of pediatric product development in the US (25). In July 2020, the FDA issued final guidance which provides the current thinking of the FDA regarding implementation of the requirement for sponsors to submit an initial pediatric study plan (iPSP), described in section 505B(e) of the Federal Food, Drug, and Cosmetic Act. In addition to a template that is recommended to be used for an iPSP, this guidance provides recommendations regarding content and timing of an iPSP submission and the content and timing of a requested amendment to an agreed iPSP. In May 2023, the FDA issued two draft guidances which, once finalized, will provide FDA's current thinking regarding the development of drugs and biological products for pediatric patients under PREA and/or BPCA (26). The draft guidance entitled, “Pediatric Drug Development Under the Pediatric Research Equity Act and the Best Pharmaceuticals for Children Act: Scientific Considerations” is intended to assist industry in developing data and obtaining information needed to support approval of drug products in pediatric populations and the draft guidance entitled “Pediatric Drug Development: Regulatory Considerations—Complying With the Pediatric Research Equity Act and Qualifying for Pediatric Exclusivity Under the Best Pharmaceuticals for Children Act” is intended to assist industry developing drug products to comply with the pediatric study requirements under PREA, and to describe the process for qualifying for pediatric exclusivity and the protections that pediatric exclusivity offers under BPCA. A complete list of FDA guidances can be found by searching FDA's website (27).

In its effort to support innovative approaches to clinical trials that are designed to improve the efficiency of drug development and regulatory decision making, FDA Center for Drug Evaluation and Research (CDER) launched a Center for Clinical Trial Innovation (C3TI) central hub in April 2024 (28, 29). C3TI will serve as a central hub to communicate and collaborate with external parties about innovative clinical trials and expand opportunities for sponsors of innovative clinical trials to interact with CDER staff, in addition to other functions. In July 2024, CDER and the Center for Biologics Evaluation and Research (CBER) established the Rare Disease Innovation Hub, a new model within the FDA designed to greatly enhance collaboration and connectivity across FDA centers to help facilitate development of treatments for rare diseases (30). The Rare Disease Innovation Hub will serve as an important new model for inter-center connectivity on cross-cutting rare disease activities and workstreams that can serve as a forum for consideration of common scientific, clinical, and policy issues related to rare disease product development. The Rare Disease Innovation Hub will also work to streamline communications with the rare disease community on issues across CDER and CBER.

The Oncology Center of Excellence, established in 2017, houses the Pediatric Oncology Program (31). The Program's mission is to promote the development of safe and effective new drugs and biologics to treat cancer in children. The Program recommends that new Pediatric Investigation Plans (PIPs) and iPSPs for new cancer products be submitted to their respective agencies simultaneously to promote global coordination and international research collaboration and encourages sponsors to seek preliminary scientific advice from both the EMA and the FDA on PIPs and iPSPs through the Pediatric Cluster Calls coordinated by the FDA's Office of Pediatric Therapeutics (December 2020).

#### 3.4.5 Japan

The Pharmaceuticals and Medical Devices Acts and Ordinance on Standards for Conduct of Clinical Trials (GCP) specify the sponsor's responsibility for submitting a notification of the clinical trial plan in advance. They also outline the requirements that a sponsor must comply when requesting an institution to conduct a CT (32, 33). Before starting a CT to obtain regulatory approval, the sponsor must submit a clinical trial notification to the Minister of Health Labor and Welfare (MHLW). A CT notification consists of non-clinical data such as chemistry, manufacturing, and controls (CMC), toxicological and pharmacological studies, and the planed CT protocol. For first-in-human trials involving a new active ingredient, a new route of administration or a new fixed combination product, the notification must be submitted at least 31 days before the scheduled start of the trial. For trials other than above, the notification must be submitted at least 2 weeks before the scheduled start of the study. The Pharmaceuticals and Medical Devices Agency (PMDA) reviews the protocol within 30 or 14 days respectively, based on collected data such as non-clinical data to ensure subject protection. PMDA submits the review result to the MHLW. If any issues regarding subject protection are detected, the MHLW can suspend the start of the trial. However, most of sponsors utilized the scientific advice system before submitting a CT notification. As a result, most notifications do not identify problems and the CT starts 30 or 14 days after notification is submitted.

#### 3.4.6 Australia

Clinical trials conducted in Australia are subject to various regulatory controls to ensure the safety of participants. The Therapeutic Goods Administration (TGA) administers the Clinical Trial Notification (CTN) and Clinical Trial Approval (CTA) schemes that allow access to “unapproved” therapeutic goods in Australian clinical trials. The TGA also undertakes inspections of clinical trial sites to check compliance with regulatory requirements.

Most clinical trials in Australia are notified to the TGA through the rapid CTN pathway, which does not involve the evaluation of data by the TGA. Human Research Ethics Committees (HRECs) are responsible for review and approval of the clinical trial. The CTA pathway is mandatory for clinical trials of high risk biologicals, and involves the evaluation of safety aspects by the TGA. Human Research Ethics Committees (HRECs) are responsible for the review and approval of other aspects of the clinical trial, including the trial protocol. Specific pediatric registration is required for HRECs to approve pediatric studies (34).

The Australian government is currently developing a single point of HREC approval and site oversight, the National One Stop Shop, with the aim of dramatically reducing the administrative burden for researchers and sponsors (35). The TGA, like many other regulators, has adopted the ICH E11(R1) guideline on clinical investigation of medicinal products in the pediatric population (36).

## 4 Discussion

By gathering up-to-date information from the CAs using a coordinated approach and an identical data collection form, this study is an effort to improve understanding of the regulatory requirements for CTs across six large jurisdictions. Additional information on jurisdiction-specific legislation and guidance material can also be found on the websites of the six Regulators. In contrast to ethics submission and review, and despite some differences in processes and timelines, approaches taken by each jurisdiction are aligned. In the USA, circumstances where a CTA/IND for an investigational medicinal product is not required are generally rare (37). Most jurisdictions have specific legislation to provide incentives and obligations for pediatric drug development (EU, US, UK) or are preparing such legislation; however, in the six jurisdictions participating in this study, in general, no difference was found in processes for CTA submission, review, and authorization for pediatric CTs compared to CTs undertaken in adult populations. However, there are additional EC/IRB requirements for CTs including children (11).

In order to support global development of medicines for children, the Pediatric Cluster was established in 2007 to promote further international alignment of pediatric CTs. It includes CAs from the United States (FDA), the European Union (EMA), Japan (PMDA), Canada (Health Canada), and Australia (TGA) (38). The Pediatric Cluster is a forum for international regulators with confidentiality commitments to participate in an informal exchange of scientific, regulatory, and ethical information and engage in discussions, primarily through monthly and *ad-hoc* teleconferences. The goal of the Pediatric Cluster is to facilitate harmonization of pediatric development plans and trial requirements and address any real or perceived divergences between the CAs. Discussions at the Pediatric Cluster consider current CT regulations in each jurisdiction and cover topics such as trial design and feasibility issues, appropriateness of pediatric extrapolation based on pharmacokinetic information, proposed study population including the minimum age for study, proposed endpoints and statistical and analytical methodologies. Efforts are made to align pediatric development programs to ensure that pediatric CTs are efficient and based on current scientific and clinical evidence. This helps to clarify whether divergences between regulators are genuine or merely perceived. In addition, the data can serve to ensure that trials are optimally designed to support pediatric marketing applications for medicines, including establishing specific labeling for pediatric indications and uses.

The Pediatric Cluster communicates with companies developing medicinal products for children in two main ways: issuing Common Commentary documents (since October 2012) and communication of high-level action items (since July 2018). Common Commentaries pertain to products with pediatric development plans that have been submitted to both FDA and EMA, are under review by both Agencies, and have been discussed at the Pediatric Cluster (38). These commentaries can be either product specific or related to a general topic.

When product specific, Common Commentaries provide companies with informal, non-binding comments on issues discussed at the Pediatric Cluster. They clarify areas where the EMA and FDA are aligned, and where they are not, referring to the reasons and potential solutions such as changes to the trial design, patient population, or consideration of a particular endpoint. Product-specific Common Commentaries and high-level action items are communicated confidentially to companies (39, 40).

General-topic commentaries on the other hand may be made public in certain instances. An example of a public-general-topic commentary is “Submitting an initial Pediatric Study Plan (iPSP) and Pediatric Investigation Plan (PIP) for the prevention and treatment of COVID19” (39, 40).

In addition to collaboration between regulators as outlined above, regulatory harmonization is proceeding via each jurisdiction's participation in the International Council for Harmonization (ICH) of Technical Requirements for Pharmaceuticals for Human Use. The first guideline in the pediatric area ‘Clinical Investigation of Medicinal Products in the Pediatric Population' (ICH E11) was published in 2000 (41). In 2017, a revised version of the guideline, which included a new Addendum [ICH E11 (R1)] was released (42). These guidelines aimed to accelerate clinical development in the pediatric population globally. More recently, a guideline on Pediatric Extrapolation (ICH E11A) was adopted in August 2024 (43).

In Europe, the CT Regulation has harmonized the CTA process across EU countries, but it does not contain common harmonized age specific requirements exclusively for pediatric clinical studies (i.e., legal age for independent consent, pediatric age grouping for assents etc.). Therefore, the Part II assessment in the CTIS is EU country specific with every Member State having its own national legislation and requirements for the assessment of the CTA and ethical documents.

In accordance with the proposal of the OECD guidance document on the Governance of CTs, some jurisdictions are considering implementing a risk-based approach based on the marketing status of a therapeutic agent in addition to a trial-specific approach that considers a broad spectrum of risk determinants (8). Clinical trials deemed to be in a low or intermediate risk category would be subject to less restrictive rules related to labeling, monitoring, regulatory content of the master file, and traceability of investigational products once approved. For instance, in some jurisdictions, it has been of a critical benefit to allow marketed drugs to be used for COVID-19 CTs. Bypassing the need to relabel or repackage the Investigational Medical Product has made the collection of data through a CT significantly cost-effective and logistically easier without compromising patient safety. Such changes could have major implications for pediatric investigator-initiated CTs studying unapproved uses of approved drugs, also known as “off-label” or repurposed drugs. The conduct of CTs falling under the OECD proposed B category (“marketed products, tested outside of marketing authorization, and supported by published evidence/established medicinal practice or new use, not supported by published evidence/established medical practice”) would be facilitated by reducing the administrative burden on sponsors without adversely affecting patient safety.

Pediatric networks, including the national network members of the Enpr-EMA Working Group on International Collaborations, play a key role in the implementation and conduct of CTs ensuring that children around the world have access to optimally designed clinical trials (44). Trials are more efficient (faster enrollment, higher numbers, adequate broader age-range) and easier to conduct internationally when there is consistency across sites and jurisdictions (45). Several network members of the WG are collaborating on pediatric site standards in and across jurisdictions to determine a minimum set of criteria required for a site be deemed competent in conducting industry or publicly funded CTs (46, 47). Collaboration among the networks has also allowed for the development of expert groups with clinical and methodological expertise to assist in the development and feasibility of CTs accessible to sponsors (46, 47). Lastly, the various networks have developed strategies and service offerings by experienced individuals to assist in the timely startup of a CT. For example, some networks are supporting academic investigators by offering CTA development and submission services by individuals who are knowledgeable of the regulatory requirements for their respective jurisdiction. Through their interactions with their respective CA, national pediatric networks can therefore play an important role in facilitating the regulatory submission of CTA. The pediatric networks are working together to identify regulatory administrative challenges and potential solutions with the aim of reducing duplication of efforts and working with academic institutions, public partners and industry to increase effective therapeutic development.

## Data Availability

The original contributions presented in the study are included in the article/Supplementary material, further inquiries can be directed to the corresponding author.

## References

[1] Joseph PD Craig JC Caldwell PH. Clinical trials in children. Br J Clin Pharmacol. (2015) 79:357–69. 10.1111/bcp.12305 24325152 PMC4345947

[2] Shakhnovich V Hornik CP Kearns GL Weigel J Abdel-Rahman SM. How to conduct clinical trials in children: a tutorial. Clin Transl Sci. (2019) 12:218–30. 10.1111/cts.12615 30657253 PMC6510379

[3] Momper JD Mulugeta Y Buckart GJ. Failed pediatric drug development trials. Clin Pharmacol Ther. (2015) 98:245–51. 10.1002/cpt.142 25963725

[4] Council of Canadian Academies. Improving Medicines for Children in Canada. The Expert Panel on Therapeutic Products for Infants, Children, and Youth (2014). Available online at: https://rapports-cac.ca/wp-content/uploads/2018/10/therapeutics_fullreporten.pdf (accessed September 01, 2024).

[5] Ward RM Benjamin DK Davis JM Gorman RL Kauffman R Kearns GL. The need for pediatric drug development. J Pediatrics. (2018) 192:13–28. 10.1016/j.jpeds.2017.08.011 28942898 PMC7942021

[6] European Union. Regulation (EU) No 536/2014 of the European Parliament and of the Council of 16 April 2014 on Clinical Trials on Medicinal Products for Human Use, Repealing Directive 2001/20/EC. Available online at: https://eur-lex.europa.eu/legal-content/EN/TXT/PDF/?uri=CELEX:02014R0536-20221205 (accessed October 2024).

[7] Biswal S Jose VM. Chapter 47: clinical trial application. In: An Overview of Clinical Trial Operation: Fundamentals of Clinical Trial Planning and Management in Drug Development Edition 2, Independently published (2018). p. 142–5.

[8] OECD. Recommendation of the Council on the Governance of Clinical Trials (2012). Available online at: https://legalinstruments.oecd.org/en/instruments/OECD-LEGAL-0397 (accessed September 17, 2024).

[9] Agreement on the European Economic Area. European Commission (2024). Available online at: https://competition-policy.ec.europa.eu/international-relations/legislation/european-economic-area_en (accessed September 11, 2024).

[10] EU Clinical Trial Regulation. Clinical Trial Information System (CTIS) for Authorities (2024). Available online at: https://euclinicaltrials.eu/ctis-for-authorities/ (accessed November 20, 2024).

[11] Stewart B Lepola P Egger GF Ali F Allen AJ Croker AK. Requirements for drug trials across six jurisdictions and special considerations for trials with children: 2. ethics review in the regulatory approval process. Front Med. (2024) 12:1539787. 10.3389/fmed.2025.1539787 40007583 PMC11850317

[12] European Union. Regulation (EC) No 1901/2006 of the European Parliament and of the Council of 12 December 2006 on Medicinal Products for Paediatric Use and Amending Regulation (EEC) No 1768/92, Directive 2001/20/EC, Directive 2001/83/EC and Regulation (EC) No 726/2004. Official Journal of the European Communities L 378/1. Available online at: http://data.europa.eu/eli/reg/2006/1901/oj (accessed January 28, 2019).

[13] European Medicines Agency. Guidance for Stepwise PIP Pilot (2022). Available online at: https://www.ema.europa.eu/en/documents/regulatory-procedural-guideline/guidance-stepwise-pip-pilot_en.pdf (accessed February 6, 2025).

[14] European Commission. Commission Proposal for the Pharmaceutical Regulation (2023). Available online at: https://health.ec.europa.eu/publications/proposal-regulation-laying-down-union-procedures-authorisation-and-supervision-medicinal-products_en (accessed February 2025).

[15] European Medicines Agency. Accelerating Clinical Trials in the EU (ACT EU): for Better Clinical Trials that Address Patients' Needs (2022). Available online at: https://www.ema.europa.eu/en/human-regulatory-overview/research-development/clinical-trials-human-medicines/accelerating-clinical-trials-eu-act-eu (accessed September 2024).

[16] Government of Canada. Clinical Trials Modernization: Consultation Paper (2021). Available online at: https://www.canada.ca/en/health-canada/programs/consultation-clinical-trials-regulatory-modernization-initiative/document.html (accessed September 01, 2024).

[17] Government of Canada. Notice to Stakeholders: Statement on the Investigational Use of Marketed Drugs in Clinical Trials (2021). Available online at: https://www.canada.ca/en/health-canada/services/drugs-health-products/drug-products/announcements/notice-statement-investigational-use-marketed-drugs-clinical-trials.html (accessed September 01, 2024).

[18] Government of UK. Consultation on Proposals for Legislative Changes for Clinical Trials (2023). Available online at: https://www.gov.uk/government/consultations/consultation-on-proposals-for-legislative-changes-for-clinical-trials (accessed September 2024).

[19] Thomas C. Saving and Improving Lifes: The Future of UK Clinical Research Delivery. Government of UK. (2021). Available online at: https://www.gov.uk/government/publications/the-future-of-uk-clinical-research-delivery/saving-and-improving-lives-the-future-of-uk-clinical-research-delivery (accessed September 2024).

[20] Health Research Authority. Approval Times for Clinical Trials Halved with Combined Review (2022). Available online at: https://www.hra.nhs.uk/about-us/news-updates/approval-times-clinical-trials-halved-combined-review/ (accessed September 2024).

[21] Government of the United Kingdom. MHRA to Streamline Clinical Trial Approvals in Biggest Overhaul of Trial Regulation in 20 Years (2024). Available online at: https://www.gov.uk/government/news/mhra-to-streamline-clinical-trial-approvals-in-biggest-overhaul-of-trial-regulation-in-20-years (accessed November 2024).

[22] Government of the United Kingdom. Patients, the NHS and the Life Sciences Sector Set to Benefit from New Clinical Trials Framework Being Laid in Parliament Today (2024). Available online at: https://www.gov.uk/government/news/patients-the-nhs-and-the-life-sciences-sector-set-to-benefit-from-new-clinical-trials-framework-being-laid-in-parliament-today (accessed January 13, 2025).

[23] U.S. Food and Drug Administration. Investigational New Drug (IND) Application. Silver Spring (MD): FDA (2023). Available online at: https://www.fda.gov/drugs/types-applications/investigational-new-drug-ind-application (accessed November 2024).

[24] U.S. Food and Drug Administration. IND Application Procedures: Exemptions and IND Requirements. Silver Spring (MD): FDA (2023). Available online at: https://www.fda.gov/drugs/investigational-new-drug-ind-application/ind-application-procedures-exemptions-ind-requirements (accessed November 2024).

[25] U.S. Food and Drug Administration. Pediatric Study Plans: Content of and Process for Submitting Initial Pediatric Study Plans and Amended Initial Pediatric Study Plans. Silver Spring (MD): FDA. (2020). Available online at: https://www.fda.gov/regulatory-information/search-fda-guidance-documents/pediatric-study-plans-content-and-process-submitting-initial-pediatric-study-plans-and-amended (accessed November 2024).

[26] U.S. Food and Drug Administration. Pediatric Drug Development Under the Pediatric Research Equity Act and Best Pharmaceuticals for Children Act. Silver Spring (MD): FDA (2023). Available online at: https://www.fda.gov/regulatory-information/search-fda-guidance-documents/pediatric-drug-development-under-pediatric-research-equity-act-and-best-pharmaceuticals-children-act (accessed November 2024).

[27] U.S. Food and Drug Administration. Regulatory Information: Search FDA Guidance Documents. Silver Spring: U.S. Food and Drug Administration (2024). Available online at: https://www.fda.gov/regulatory-information/search-fda-guidance-documents (accessed November 2024).

[28] U.S. Food and Drug Administration. CDER Center for Clinical Trial Innovation (C3TI). Silver Spring (MD): FDA (2024). Available online at: https://www.federalregister.gov/documents/2024/04/16/2024-07829/center-for-drug-evaluation-and-research-center-for-clinical-trial-innovation (accessed November 2024).

[29] U.S. Food and Drug Administration. CDER - Center for Clinical Trial Innovation (C3Ti) [Internet]. Silver Spring (MD): U.S. Food and Drug Administration. (2024). Available online at: https://www.fda.gov/about-fda/center-drug-evaluation-and-research-cder/cder-center-clinical-trial-innovation-c3ti (accessed November 2024).

[30] U.S. Food and Drug Administration. FDA Takes Exciting Steps Toward Establishing the Rare Disease Innovation Hub. Silver Spring (MD): FDA (2023). Available online at: https://www.fda.gov/news-events/fda-voices/fda-takes-exciting-steps-toward-establishing-rare-disease-innovation-hub (accessed November 2024).

[31] Reaman G Karres D Ligas F Lesa G Casey D Ehrlich L. Accelerating the global development of pediatric cancer drugs: a call to coordinate the submissions of pediatric investigation plans and pediatric study plans to the European Medicines Agency and US Food and Drug Administration. J Clin Oncol. (2020) 38:4215–25. 10.1200/JCO.20.02152 32946356

[32] Japanese Law Translation. Act on Securing Quality, Efficacy and Quality of Products including Pharmaceutical and Medical Devices. (2015). Available online at: https://www.japaneselawtranslation.go.jp/en/laws/view/3213 (accessed September 17, 2024).

[33] Good Clinical Practice (GCP). Ordinance on Standards for Conduct of Clinical Trials (GCP) (MHW Ordinance No. 28 dated March 27, 1997, partially revised by MHLW Ordinance No. 9 dated January 22, 2016). Available online at: https://www.pmda.go.jp/files/000152996.pdf (accessed September 2024).

[34] Therapeutic Goods Administration. Clinical Trials. Therapeutic Goods Administration (2024). Available online at: https://www.tga.gov.au/clinical-trials (accessed September 2024).

[35] Australian Commission on Safety and Quality in Health Care. National One-Stop Shop: National Platform for Health-Related Human Research (2024). Available from: https://www.safetyandquality.gov.au/our-work/health-and-human-research/national-one-stop-shop-national-platform-health-related-human-research (accessed November 2024).

[36] Therapeutic Goods Administration. ICH E11(R1) Guideline on Clinical Investigation of Medicinal Products in the Pediatric Population: Addendum. The Netherlands: European Medicines Agency Amsterdam (2023).

[37] U.S. Food and Drug Administration. Investigational New Drug Applications (INDs)—Determining Whether Human Research Studies can be Conducted Without an IND. Silver Spring (MD): FDA. (2020). Available from: https://www.fda.gov/regulatory-information/search-fda-guidance-documents/investigational-new-drug-applications-inds-determining-whether-human-research-studies-can-be (accessed November 2024).

[38] Penkov D Tomasi P Eichler I Murphy D Yao LP Temeck J. Pediatric medicine development: an overview and comparison of regulatory processes in the European Union and United States. Therap Innov Regul Sci. (2017) 51:360–71. 10.1177/2168479017696265 28674673 PMC5493316

[39] U.S. Food and Drug Administration. FDA/EMA Common Commentary on Submitting an Initial Pediatric Study Plan (iPSP) and Pediatric Investigation Plan (PIP) for the prevention and treatment of COVID-19 (2021). Available online at: https://www.fda.gov/media/138489/download (accessed Oct 2024).

[40] US Food and Drug Administration. International Collaboration—Pediatric Cluster (2024). Available online at: https://www.fda.gov/science-research/pediatrics/international-collaboration-pediatric-cluster#common-comentary-1 (accessed September 2024).

[41] Scientific Guideline. ICH E11 (R1) Step 5 Guidelines on Clinical Investigation of Medicinal Products in the Pediatric Population (2017). Available online at: https://www.ema.europa.eu/en/ich-e11r1-step-5-guideline-clinical-investigation-medicinal-products-pediatric-population-scientific-guideline (accessed September 2024).

[42] ICH Harmonized Guidelines. Addendum to ICH E11: Clinical Investigation of Medicinal Products in the Pediatric Population (2017). Available online at: https://database.ich.org/sites/default/files/E11_R1_Addendum.pdf (accessed September 2024).

[43] ICH Harmonized Guidelines. Pediatric Extrapolation E11A (2024). Available online at: https://www.ich.org/news/ich-e11a-guideline-reaches-step-4-ich-process (accessed October 2024).

[44] Lepola P Tansey S Dicks P Preston J Dehlinger-Kremer M. Pharmaceutical industry and paediatric clinical trial networks in Europe—How do they communicate? Appl Clin Trials. (2016) 6:697–705. 10.1007/s00103-019-02952-8 31069417

[45] Greenberg RG McCune S Attar S Hovinga C Stewart B Lacaze-Masmonteil T. Pediatric clinical research networks: role in accelerating development of therapeutics in children. Therap Innov Regulat Sci. (2022) 56:934–47. 10.1007/s43441-022-00453-6 36085251 PMC9462608

[46] Siapkara A Fracaso C Egger GF Rizzari C Trasorras CS Athanasiou D. Recommendations by the European Network of Paediatric Research at the European Medicines Agency (Enpr-EMA) Working Group on preparedness of clinical trials about paediatric medicines progress. Arch Dis Child. (2021) 106:1149–54. 10.1136/archdischild-2020-321433 33858819 PMC8666697

[47] Attar S Price A Hovinga C Stewart B Lacaze-Masmonteil T Bonifazi F. Harmonizing quality improvement metrics across global trials networks to advance paediatric clinical trials delivery. Therap Innov Regul Sci. (2024) 58:953–64. 10.1007/s43441-024-00663-0 38902577 PMC11335960

