# Supplementary material for: Requirements and special considerations for drug trials with children across six jurisdictions: 1. Clinical trial application review in the regulatory approval process
Source: Front Med (Lausanne). 2025 Apr 15;12:1542408. doi: 10.3389/fmed.2025.1542408 (PMC12037545; doi:10.3389/fmed.2025.1542408)
Supplement: Supplementary file 1 [file Data_Sheet_1.pdf]

ENVIRONMENTAL SCAN CLINICAL TRIAL APPLICATIONS IN: AUSTRALIA CANADA EU, JAPAN, AND USA,

Version – 29April, 2019

*** Please complete the below questions within the Word document. The MICYRN team will compile the completed documents. The intention is for the complied document to be presented in a white paper format. Please provide citations and references for responses if possible.*

Glossary:

Clinical Trial Application: comprehensive information application about the investigational medicinal product(s)/medical device and planned clinical trial, enabling regulatory authorities to assess the acceptability of conducting a study. The regulatory authorities' assessment covers the investigational medicinal product/device properties, the benefit/risk ratio of the study, the quality of the information provided to the trial subjects, and the suitability of the clinical sites and investigators.

Jurisdiction: the territory or region that governs the authority of clinical trial conduct and requirements.

Questions highlighted in blue are questions to be answered by the networks specifically

Objectives:

- To assess the current regulatory review and ethics requirements for pediatric studies in Australia, Canada, EU, Japan and the US.
- To identify the key similarities and differences between these jurisdictions that impact the feasibility and practicality of conducting international pediatric trials

Questions are grouped under 3 categories

1. Clinical Trial Requirements
2. Pediatric Clinical Trial Review Process
3. Ethics

1) CATEGORY 1: CLINICAL TRIAL REQUIRMENTS

a) Is there specific legislation regarding conduct of clinical trials in children within your jurisdiction? If so, briefly outline

   i) Is the legislation the same for medical devices as for medications?

b) Is this legislation proposed to change in the near future? If so, what is the new proposed legislation?

c) Are there differences in CTA submission requirements depending on the type of sponsor (e.g. industry versus academic sponsor)? If yes, please describe.

d) How is the investigational status of a drug determined in your country?

   In this context, the "investigational status" refers to the off-label use of medications, investigational new drugs and devices etc.

e) What is the definition of a clinical trial in your jurisdiction?

f) The OECD Recommendation on the Governance of Clinical Trials currently stratifies the oversight requirements for clinical trials as outlined below. Currently, is a CTA required for pediatric trials within your jurisdiction for the following:

i) Non-authorized medications (investigational new drugs or devices)?

ii) Authorized medications used outside the parameters of marketing authorization (e.g. different patient population, dosage, route of administration indication, etc.)?

(1) Not supported by established medical practice

(2) Supported by established medical practice

iii) Authorized medications tested within marketing authorization?

g) Are there circumstances when Sponsors do *not* require regulatory approval for pediatric clinical trial?

i) Are there clinical studies that would automatically get an exemption and therefore not need to be sent for review?

ii) What are the criteria for an exemption (e.g. design- risk level- standard of care– clinical development phase etc.)? Are other applications screened for exemption eligibility?

h) Are CTAs required for natural health product studies (e.g. vitamins, homeopathic medicines, herbal remedies)?

i) Who decides if a CTA is required for a pediatric trial (e.g. ethics board, committee, regulatory authority)?

2) PEDIATRIC CLINICAL TRIAL REVIEW PROCESS

a) Who reviews the CTA/Where is the CTA sent to within your jurisdiction?

i) Are there multiple directorates or regulatory review groups that are responsible for CTA review and approval?

b) When a clinical trial involves a combination of biological agent, medical devices or pharmaceutical agent, how are the various components reviewed within your jurisdiction?

c) Please describe any differences in the review process if an adult study includes a pediatric subgroup or a pediatric study includes an adult subgroup.

d) Is there a default approval timeframe from the date of receipt of the completed application? When does the default period start (e.g. when the application is received, after administrative review/screening of the application)?

   i) Do all regulatory review groups have the same turnaround times?

   ii) Is an acknowledgement letter sent to the sponsor to indicate the start of the review period?

   iii) Are there mechanisms to shorten the review timeline?

e) During the review process, how does the sponsor resolve issues identified/questions posed by the regulatory body(ies)?

   i) Are requests for additional information received more than once and from multiple areas within the applicable regulatory review group (e.g. from protocol reviewers and/or product quality assessors)?

   ii) How long does the sponsor have to provide the requested information/make any changes to the clinical trial application package?

iii) What is the average length of time to receive regulatory approval after responding to such requests so that a clinical trial can commence?

iv) Are these requests, and response times, consistent within all organizational groups?

f) Please indicate the possible responses issued by the regulatory group after a CTA is submitted.

Examples include: No Objection Letter, green light approval is assumed if no regulatory response received within a certain timeframe etc.

g) Are there other documents that the sponsor must complete following the regulatory approval of a CTA, prior to trial commencement (e.g. clinical trial site information forms, REB attestation letter, fax-back forms)?

h) Are there required or potential post-authorization ongoing obligations mandated by the regulator body(ies)? If so, please list.

i) Do changes made to the protocol require the filing of a CTA-Amendment? Is there a provision for a Notification Letter to be sent instead based on the changes? If yes, what are the criteria to determine which communication is applicable?

3) Ethics (EC: Ethics Committee (EU, Japan), REB: Research Ethics Board (Canada), IRB: Institutional Review Board (US), Human Research Ethics Committee (Australia))

a) Does your jurisdiction **currently** allow a centralized (or single) ethics board review? If yes, does this apply to:

i) Academic (investigator initiated) clinical trials, please describe the process?

ii) Industry sponsored clinical trials, please describe the process?

iii) If your jurisdiction does not have a centralized ethics board process, do you have an ethics harmonization process (e.g. approval in one country/region allows for an expedited review in another region/country)?

b) Does your jurisdiction allow the use of a for-profit or commercial ethics board? In which circumstances?

c) Is the ethics review process the same for a publicly funded clinical trial as it is for privately funded clinical trials? If not, please describe how they differ.

d) In addition to a central ethics board, does your jurisdiction also require local ethics board approval/review for a clinical trial?

   i) If yes, what needs to be approved by the local ethics board?

e) Does your jurisdiction require ethics committees/ethics board review of clinical trials in children to have at least one member knowledgeable in pediatrics/pediatric research?

f) Are ethics boards in your jurisdiction provided with educational material addressing ethical challenges with children and randomized controlled trials?

g) Is the CTA submission and ethics board review coordinated (Does one need to be submitted before the other or are they simultaneously submitted)? If yes, please, describe.

h) Is there a proposed change in the near future for the ethics review process in your jurisdiction? If yes, please describe the process.

   i) Does the proposed change differ based on the country within your jurisdiction, please describe?

i) Are there specific requirements that need to be included in an ethics submission for pediatric applications that differ from adult applications?

   When is an assent form required for pediatric ethics submissions?

j) Do the informed consent or assent forms need to be signed by both parents/caregivers?

    i) Is a dual signature dependent on the risk/benefit of the study?
